# Ganglion Cyst at the Lateral Aspect of the First Metatarsophalangeal Joint: Dermatological Management With Punch Biopsy Excision

**DOI:** 10.7759/cureus.80987

**Published:** 2025-03-22

**Authors:** Abdulrahman Saleh Aldairi, Faris Alsaedi, Yusra Bundagji, Rana Al-Zaidi, Homaid Alotaibi

**Affiliations:** 1 Department of Dermatology, King Faisal Hospital, Ministry of Health, Makkah, SAU; 2 Department of Anatomic Pathology, King Faisal Hospital, Ministry of Health, Makkah, SAU

**Keywords:** dermatology, dermatosurgery, first metatarsophalangeal joint, foot ganglion cyst, foot mass, ganglion cyst, joint capsule, metatarsal bone, punch biopsy, skin punch biopsy

## Abstract

Ganglion cysts are prevalent benign lesions originating from joint capsules or tendon sheaths, predominantly found in the wrist, while their presence on the toes is extremely rare. We report the case of a 27-year-old female who presented with a painless, mobile, rubbery nodule on the lateral aspect of the first metatarsal of the right foot. Generally, ganglion cysts in the foot are uncommon, and their development is thought to be associated with repetitive mechanical stress, leading to mucoid degeneration of collagen tissue. While conservative treatments such as aspiration and steroid injections exist, they have a high recurrence rate, making surgical excision the preferred option. This case highlights an unusual presentation of a ganglion cyst on the lateral aspect of the first metatarsal and emphasizes the importance of considering mechanical stress as a contributing factor. Punch biopsy excision provided a definitive, minimally invasive, and effective treatment, demonstrating excellent results with no recurrence noted during follow-up*.*

## Introduction

Ganglion cysts occur frequently as benign cystic formations arising from connective tissues, such as joint capsules and tendon sheaths, with less frequent occurrence within bone [1]. However, ganglion cysts affecting the toes are extremely rare, accounting for only 2% of cases [2,3]. These cysts typically contain a gelatinous substance, and the majority are asymptomatic [4]. Surgical excision demonstrates a markedly reduced recurrence rate in comparison to conservative therapy [5]. Recurrence is frequently ascribed to the inadequate excision of the cyst wall [6]. Here, we present a case of a young woman with the unusual site of a ganglion cyst located on the lateral aspect of the big toe, which was successfully excised using a punch biopsy.

## Case presentation

**Figure 1:**
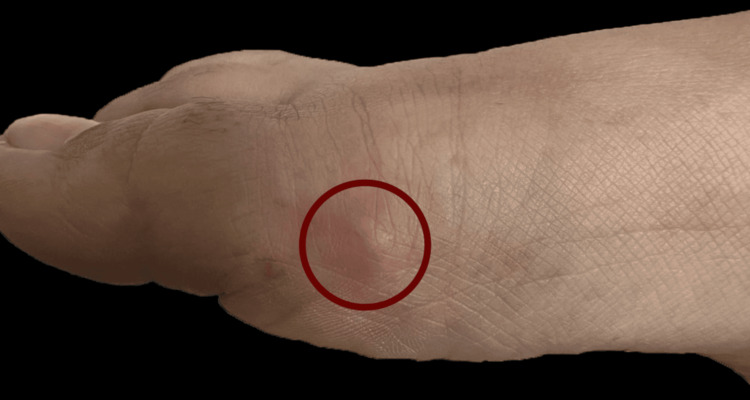
A nodule on the lateral side of the first metatarsal area, with slight erythema of the overlying skin. The area is marked with a red circular shape for identification.

A 27-year-old female, with no significant previous medical history, presented to the outpatient clinic with a complaint of a painless mass on the outer side of her right foot (Figure 1), which had been present for the past six months. She reported that the mass partially disappears when she moves her toe and mentioned prolonged use of tight shoes.

**Figure 2:**
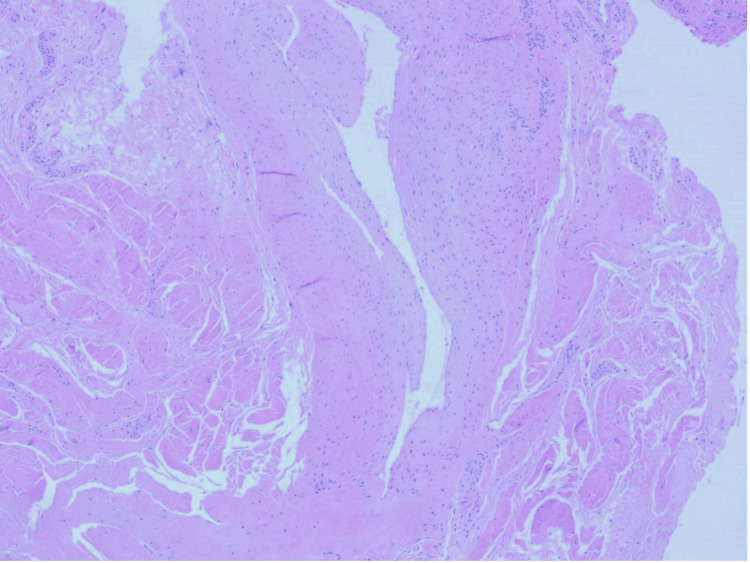
Low-power view of a ganglion cyst showing a unilocular collapsed cystic structure comprised of a dense collagenous wall (hematoxylin-eosin stain, original magnification x40).

**Figure 3:**
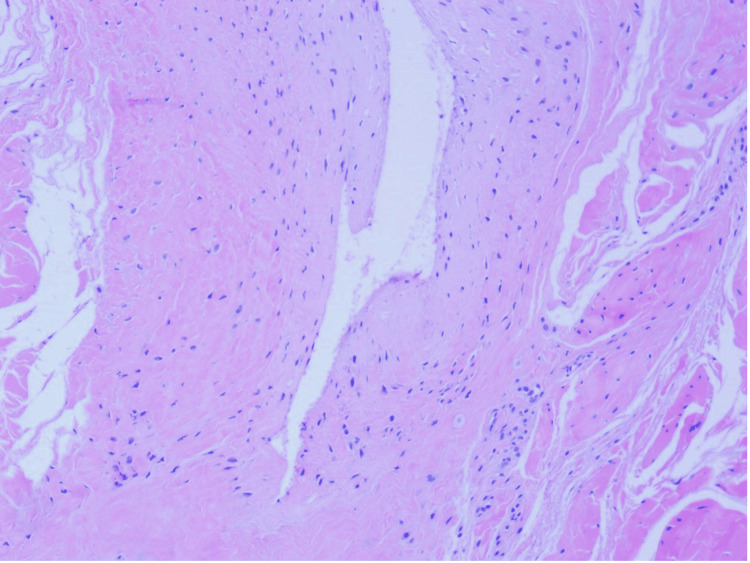
Ganglion cyst showing a hypocellular dense collagenous wall that is devoid of true epithelial lining (hematoxylin-eosin stain, original magnification x100).

A primary care physician ordered an X-ray, but the cause of the mass was not identified. She was then referred to the dermatology clinic. Upon physical examination, a 1 x 1 cm nodule was noted on the lateral aspect of the first metatarsal. The overlying skin appeared slightly red, and the nodule was soft, mobile, and not attached to underlying structures. It had a rubbery consistency and was non-tender. Foot and ankle movements were normal, and the neurovascular exam was intact. Based on these findings, a ganglion cyst was highly suspected. Due to the small size of the mass, it was decided to excise it in the minor dermatological procedures room. The procedure was performed under sterile technique using 1% lidocaine as local anesthesia. A single 4 mm punch biopsy tool was then used to completely excise the cyst, ensuring that the entire capsule was removed, rather than merely puncturing and aspirating its contents. The cyst was gently elevated using forceps, and careful blunt dissection was performed around the margins to ensure intact excision. The extraction technique was similar to a scoop-out method, in which the cyst was carefully lifted and separated from the surrounding tissue without rupture. The biopsy site was closed with a primary closure technique using interrupted sutures to optimize healing. The extracted specimen was sent for pathology, which confirmed the diagnosis of a ganglion cyst (Figures 2-3).​​​​​​​

At follow-up visits at six months and one year, the patient returned without recurrence of the mass at the previously affected site. She was advised to avoid wearing tight shoes and was discharged from the dermatology clinic with complete recovery.

## Discussion

Ganglion cysts are synovial cysts filled with mucus, predominantly found in the wrist, and are often linked to trauma, mucoid degeneration, or synovial herniation [7]. They predominantly occur in the upper extremities and are less frequently found in the feet and ankles [7]. In the foot, ganglion cysts are typically located around the ankle joint and the dorsum of the midtarsal region [8]. Approximately 75% of biopsy-confirmed soft tissue masses in the foot are benign tumors or non-tumorous lesions, including several histological subtypes [9].

In the present case, the ganglion cyst originated from the lateral aspect of the first metatarsal, a highly uncommon presentation. Ganglion cysts are typically found in anatomical regions subjected to continuous mechanical stress [3]. Repetitive activity in these areas leads to mucoid degeneration of collagen tissue, resulting in the formation of amorphous gelatinous material [3]. Occupational factors contribute significantly to ganglion cyst development, particularly in individuals who engage in repetitive joint movements, such as those involving the wrist and fingers [3]. Additionally, non-occupational factors, including osteoarthritis and rheumatoid arthritis, have been linked to ganglion cyst formation [2,3].

Our patient reported frequent use of tight-fitting shoes, which likely subjected the lateral aspect of the first metatarsal to repeated minor mechanical stress, contributing to cyst development. The differential diagnosis encompassed rheumatoid nodule, acquired fibrokeratoma, periungual fibroma linked to tuberous sclerosis, acral mucinous fibrokeratoma, fibrous histiocytoma, myxoma, superficial acral fibromyxoma, superficial angiomyxoma, glomus tumor, and neurofibroma [10,11]. The diagnosis of a ganglion cyst can be established based on clinical history, physical examination, radiographic findings, and histopathological analysis following biopsy [2].

Given the lesion's small size and the absence of any personal or family history of malignancy in our patient, a direct excision via punch biopsy was performed, with subsequent histopathological confirmation of the diagnosis. Punch biopsy excision is frequently employed in dermatology for minor lesions; however, its usage for ganglion cyst excision is not widely documented. The rationale may be that the majority of ganglion cysts manifest on the wrist or ankle, where more extensive excision methods are favored due to their closer connection to joint structures.

On the other hand, minor cysts in regions with minimal underlying joint involvement, such as the lateral metatarsophalangeal (MTP) joint, can be efficiently addressed using the punch biopsy technique. This case report adds to the growing evidence that punch biopsy excision may serve as an acceptable alternative for treating particular ganglion cysts. Anatomically, the first metatarsal bone is surrounded by key structures, including the joint capsule of the first MTP joint, as well as the extensor hallucis longus and flexor hallucis longus tendons, which insert on opposite sides of the distal phalanx of the great toe [12].

**Figure 4:**
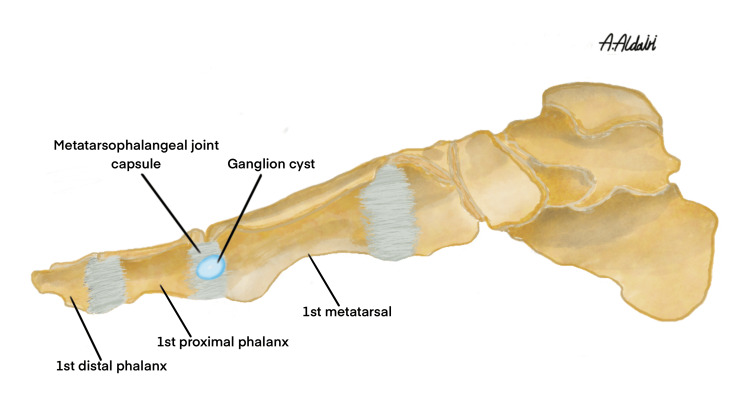
An illustration showing the exact location where the ganglion cyst originates, from the lateral side of the capsule of the first metatarsophalangeal joint. Figure Credit: Abdulrahman Saleh Aldairi

The lateral aspect of the first metatarsal head has relatively sparse tendinous and neurovascular structures. Based on surface anatomy and the distribution of surrounding anatomical components, we determined that the ganglion cyst in this case originated from the joint capsule of the first MTP joint (Figure 4).

To the best of our knowledge, this is the first reported case of a ganglion cyst originating from the lateral aspect of the first MTP joint. The management of ganglion cysts can be categorized into conservative and surgical interventions. Conservative treatments include padding, footwear modification, warm compress, aspiration, steroid injection, or a combination of aspiration with steroid injection to promote scar formation and prevent further communication with degenerative joint capsules or tendons [13]. However, recurrence rates after surgical excision range from 11% to 63% [5]. Research demonstrates that full cyst wall removal markedly diminishes the risk of recurrence compared to aspiration treatments, which exhibit a recurrence rate of approximately 62.5% [5]. Surgical excision is thus favored in cases where total removal is needed to reduce the chance of recurrence [5]. Despite its advantages, surgical excision carries risks, such as postoperative pain, stiffness, infection, scarring, and potential neurological complications, either temporary or permanent [1]. Nevertheless, surgical removal remains the gold standard for the definitive management of ganglion cysts [1].

## Conclusions

Ganglion cysts of the foot are rare, particularly on the lateral aspect of the first metatarsal region. This case highlights the significance of mechanical stress as a contributing factor to cyst formation. Given the high recurrence rate associated with conservative treatment, complete surgical excision remains the preferred approach. The successful resolution of this case, without recurrence, suggests that punch biopsy excision is a viable and effective treatment option for small ganglion cysts in atypical locations. Nevertheless, we acknowledge that an extended follow-up period of more than one year would offer a more solid confirmation of recurrence.
